# Omega-3 Docosahexaenoic Acid as a Promising Inducer of Ferroptosis: Dynamics of Action in Prostate and Colorectal Cancer Models

**DOI:** 10.1134/S160767292460132X

**Published:** 2025-01-22

**Authors:** D. M. Olkhovik, M. O. Silkina, A. V. Razumovskaya, K. V. Klycheva, A. A. Fatkulin, T. A. Kulagin, S. V. Nikulin

**Affiliations:** National Research University Higher School of Economics, Moscow, Russia

**Keywords:** ferroptosis, prostate cancer, colorectal cancer, omega-3 polyunsaturated fatty acids

## Abstract

Ferroptosis is an iron-dependent form of programmed cell death (PCD) associated with lipid membrane peroxidation. It has gained attention in cancer research because some tumor cells that are resistant to other forms of PCD are sensitive to ferroptosis. Despite the significant amount of research on ferroptosis, the list of known inducers remains limited, creating opportunities to discover new compounds with clinical potential. Recent studies have shown that long-chain polyunsaturated fatty acids, such as omega-3 docosahexaenoic acid (DHA), can function as ferroptosis inducers. In this study, we examined the kinetics of ferroptosis in prostate and colorectal cancer cells under the influence of erastin and DHA. Differences in the kinetics and mechanisms of action were observed. Moreover, cells resistant to erastin were found to be sensitive to DHA, confirming the potential of further research into its use as an anticancer agent.

## INTRODUCTION

Ferroptosis is a relatively new form of cell death, distinct from apoptosis and other types of programmed cell death (PCD) [1]. It is initiated by iron accumulation and excessive lipid peroxidation, which leads to the destruction of cell membranes [1]. In the context of cancer research, ferroptosis has recently attracted increasing interest. For example, drug-resistant tumor cells were reported to be sensitive to ferroptosis, which makes it a promising alternative for inducing tumor cell death [2]. This observation can be explained by data indicating an increased iron requirement in tumor cells compared to normal cells [3]. Thus, the study of ferroptosis in the context of cancer is a very promising direction that has only recently begun to actively develop.

Currently, there are already a large number of studies on the sensitivity of tumor cells to ferroptosis induction [4, 5]. However, there are practically no works that determine and compare the kinetics of ferroptosis between different types of tumor cells and different ferroptosis inducers. In addition, despite the fact that the list of potential ferroptosis inducers is constantly expanding, it still remains quite limited [6], thereby providing a wide field for the search for new compounds that can cause this type of cell death. For  example, according to recent data, such compounds may include, in particular, polyunsaturated fatty acids [7].

In this work, the sensitivity of two prostate cancer cell lines and two colorectal cancer lines to the classical ferroptosis inducer erastin and to omega-3 docosahexaenoic acid (DHA) was studied, since it was previously shown that the latter induces tumor cell death most effectively than other polyunsaturated fatty acids [7]. The kinetics of induced cell death was compared, and differences between the studied ferroptosis inducers were revealed.

## MATERIALS AND METHODS

Prostate cancer (PC-3, Du145) and colorectal cancer (Caco-2, HT-29) cells were cultured in DMEM/F12 medium (PanEco, Russia) containing 10% FBS (Capricorn, Germany), 1% Glutamax (Gibco, United States) and 1% Anti-anti (Gibco, United States) at 37°C and 5% CO_2_ in a cell incubator (Alphavita, China). Cell cultures were then subcultured using 0.25% trypsin–EDTA solution (PanEco, Russia) every 2–3 days. Cell growth dynamics were assessed visually using a ZOE Fluorescent Cell Imager inverted microscope (Bio-Rad, United States).

To analyze the kinetics of ferroptosis, cells were plated at a rate of 5000 cells per well in a 96-well plate and incubated in 100 μL of culture medium in a cell incubator (37°C, 5% CO_2_) for 24 h. The culture medium was then replaced with the control medium or a medium containing 10 μM erastin [8], 200 μM DHA [7], or 0.5 μM ferrostatin-1, or their combinations. Cells were incubated at 37°C and 5% CO_2_ for 48 h. To visualize cell death, the added medium also contained propidium iodide (PI) at a concentration of 1 μg/mL. Since the red fluorescent signal could only be detected after the cell membrane was disrupted, the cell death kinetics were determined by counting the number of red fluorescent objects (dead cells) over time using the IncuCyte® S3 Live Cell Analysis System (Sartorius, United States). The experiment was performed in triplicate. The statistical significance of the observed differences was assessed using the analysis of variance (ANOVA).

## RESULTS AND DISCUSSION

**Fig. 1.**
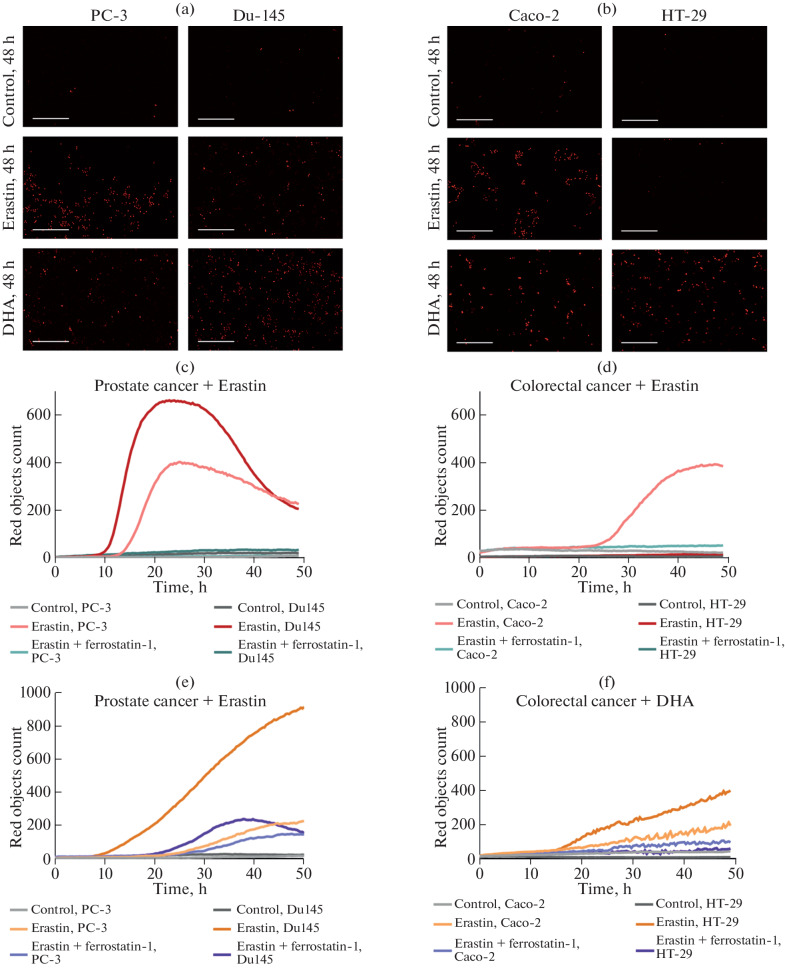
Live microphotographs of prostate cancer cell cultures (a) and colorectal cancer (b) 2 days after treatment with erastin and DHA; viability assessment results using the IncuCyte assay after treatment with erastin (10 µM) and a combination of erastin with ferrostatin-1 (0.5 µM) for prostate cancer cells (c) and colorectal cancer (d); viability assessment results using the IncuCyte assay after treatment with DHA (200 µM) and a combination of DHA with ferrostatin-1 (0.5 µM) for prostate cancer cells (e) and colorectal cancer (f).

In this work, we successfully determined the kinetics of ferroptosis in two prostate cancer cell lines and two colorectal cancer cell lines (Fig. 1). Photomicrographs of dead cells visualized with PI are shown in Figs. 1a, 1b. It was found that the prostate cell lines studied (Du145 and PC-3 in Fig. 1c) were more sensitive to erastin-induced ferroptosis than the colorectal cancer cell lines (Caco-2 and HT-29 in Fig. 1d). The Du145 cells were the most sensitive to ferroptosis, demonstrating the earliest death (approximately 8 h after treatment). Another prostate cell line, PC-3, was also susceptible to ferroptosis, but with a later onset of death (approximately 14 h after erastin treatment). The colorectal cancer cell lines demonstrated a significantly lower sensitivity. Caco-2 cells began to die approximately 26 h after treatment, whereas HT-29 cells did not show a significant increase in the number of dead cells compared to the control over 48 h.

Interestingly, all the cell lines studied were sensitive to DHA treatment. The classic ferroptosis inhibitor ferrostatin-1 also reduced the level of cells killed by DHA in all lines (Figs. 1e, 1f). In the case of the prostate cancer lines (Fig. 1e), Du145 cells again demonstrated the highest sensitivity. The death of cells began approximately 10–12 h after DHA treatment, which is somewhat later than after the corresponding treatment with erastin. The PC-3 cell line, as in the case of erastin treatment, showed lower sensitivity; however, this time cell death began noticeably later, approximately 26 h after the start of the experiment. An interesting observation was that the colorectal cancer line HT-29, which turned out to be the most resistant to the action of erastin, demonstrated a high sensitivity to DHA: cell death began already 12–14 h after treatment, and ferrostatin-1 completely protected the cells of this line from death (Fig. 1f). The effect of DHA on the Caco-2 line was similar to that on the PC-3 line (Figs. 1e, 1f).

In this work we demonstrated that different sensitivity of tumor cell lines to ferroptosis can be expressed not only in differences in viability, but also in different times of the onset of ferroptosis in cells. In particular, it was previously shown that prostate cancer cell lines PC-3 and Du145 are sensitive to erastin [5]. However, in this study we showed for the first time that, under the treatment with erastin, the death of PC-3 cells begins significantly (approximately 6 h) later than in the case of Du145 cells. Therefore, studying the process of death in dynamics can allow a more accurate determination of sensitivity than measurements at a single time point.

Studies of the kinetics of cell death under the influence of various ferroptosis inducers may also help to reveal differences in their mechanisms of action. It was previously shown that polyunsaturated fatty acids (PUFAs), including docosahexaenoic acid, can induce ferroptosis in tumor cells [7, 9]. However, other studies demonstrated that, in addition to ferroptosis, PUFAs (in particular, DHA) can also induce the apoptotic pathway of cell death [10, 11]. Therefore, their mechanism of action may be mixed. In our work, we compared the sensitivity of cells to treatment with erastin and DHA and found a number of noticeable differences. First, the rate of cell death under the influence of DHA was significantly lower than that under the influence of erastin, despite the fact that the time of the onset of cell death could be comparable. Second, the classic ferroptosis inhibitor, ferrostatin-1, completely prevented DHA-induced cell death only in HT-29 colorectal cancer cells. In all other cases, it significantly reduced the number of dead cells but could not completely stop the cell death induction. These data suggest that the mechanism of action of DHA significantly differs from that of erastin and that DHA has the potential to induce cell death not only through ferroptosis. Moreover, in this study, we showed that erastin-resistant cells can be sensitive to DHA, which confirms the prospects of further investigation of this omega-3 PUFA as an anticancer drug.
